# Genetic determinants of circulating galectin‐3 levels in patients with coronary artery disease

**DOI:** 10.1002/mgg3.1370

**Published:** 2020-06-23

**Authors:** Yu‐Huang Liao, Ming‐Sheng Teng, Jyh‐Ming J. Juang, Fu‐Tien Chiang, Leay‐Kiaw Er, Semon Wu, Yu‐Lin Ko

**Affiliations:** ^1^ Division of Endocrinology and Metabolism Department of Internal Medicine Taipei Tzu Chi Hospital Buddhist Tzu Chi Medical Foundation New Taipei city Taiwan; ^2^ Department of Research Taipei Tzu Chi Hospital Buddhist Tzu Chi Medical Foundation New Taipei city Taiwan; ^3^ Cardiovascular Center and Division of Cardiology Department of Internal Medicine National Taiwan University Hospital Taipei Taiwan; ^4^ National Taiwan University College of Medicine Taipei Taiwan; ^5^ Cardiovascular Center and Division of Cardiology Fu‐Jen Catholic University Hospital New Taipei city Taiwan; ^6^ School of Medicine Tzu Chi University Hualien Taiwan; ^7^ Department of Life Science Chinese Culture University Taipei Taiwan; ^8^ Cardiovascular Center and Division of Cardiology Department of Internal Medicine Taipei Tzu Chi Hospital Buddhist Tzu Chi Medical Foundation New Taipei city Taiwan

**Keywords:** CAD, Galectin‐3, *LGALS3* SNP

## Abstract

**Background:**

Galectin‐3 plays a crucial role in the regulation of inflammation. The aim of this study was to elucidate the association between *LGALS3* genotypes, galectin‐3 levels, and inflammatory marker levels in patients with coronary artery disease (CAD).

**Results:**

A total of 474 patients with CAD were enrolled. Significant correlations were discerned between galectin‐3 levels and leukocyte counts, C‐reactive protein, soluble intercellular adhesion molecule‐1, and matrix metalloproteinase 9 levels (all *p* < .05). The *LGALS3* rs2274273, rs4644, rs4652 genotypes, and haplotypes CAC, CCC, and ACT exhibited a significant association with galectin‐3 levels (for genotypes, *p* = 1.05 × 10^−25^, 3.54 × 10^−25^, and 2.74 × 10^−7^, respectively). Multivariate analysis showed *LGALS3* rs2274273 and rs4644 genotypes contributing to 20.8% variation of galectin‐3 levels. However, there was no association between *LGALS3* genotypes and other inflammatory marker levels.

**Conclusions:**

Our data showed strong genetic determinants of galectin‐3 levels in patients with CAD. The galectin‐3 levels, but not *LGALS3* genotypes, were associated with multiple inflammatory marker levels. Further study may be necessary to elucidate the molecular mechanism of galectin‐3 in the pathogenesis of chronic inflammatory disorders.

## Graphical abstract

Galectin‐3 levels significantly correlated with leukocyte counts, C‐reactive protein, soluble intercellular adhesion molecule‐1, and matrix metalloproteinase 9 levels while LGALS3 SNPs rs2274273 and rs4644 genotypes independently associated with and contributed to 20.8% of galectin‐3 levels in patients with coronary artery disease. The absence of a significant association between LGALS3 gene variants and the inflammation markers levels suggested that it should be cautious when galectin‐3 was used as a therapeutic target for chronic inflammatory disorders, such as coronary artery disease.

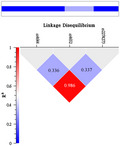

## 1 INTRODUCTION

Galectin‐3 is a beta‐galactoside‐specific lectin belonging to the galectin gene family that is localized in various parts of tissue, including the extracellular and cytoplasmic parts and the nuclear microenvironment (Henderson & Sethi, 2009). Galectin‐3 was reported to regulate numerous biological functions such as cell growth, cell adhesion, cell–cell interaction, apoptosis, angiogenesis, and mRNA processing (Califice, Castronovo, & Van Den Brule, 2004). Galectin‐3 can function as a regulatory molecule in various stages of inflammation, and manipulation of galectin‐3 expression has been demonstrated to have a major effect on inflammation (Henderson & Sethi, 2009). Human microvascular lung endothelial cells treated with galectin‐3 exhibited an increased expression of inflammatory markers, including E‐selectin and intercellular adhesion molecule‐1 (ICAM‐1) (Chen et al., 2013). In an in vitro study, extracellular galectin‐3 was determined to play a role in the induction of matrix metalloproteinase 9 (MMP9) expression (Dange, Agarwal, & Kalraiya, 2015). Increased MMP9 levels were reported to be associated with fibrinogen, an inflammation marker induced by various mediators (Wu et al., 2010).

In recent decades, galectin‐3 has come to be recognized as a potential biomarker with clinical value. Galectin‐3 levels have been associated with cancer (Califice et al., 2004), immunological disorders (Dhirapong, Lleo, Leung, Gershwin, & Liu, 2009; Henderson & Sethi, 2009), obesity (Weigert et al., 2010), and risk factors for cardiovascular disease (de Boer, van Veldhuisen, et al., 2012) and heart failure (de Boer et al., 2011). Several studies revealed a correlation between galectin‐3 levels and outcome in coronary artery disease (CAD) (Aksan et al., 2016; Maiolino et al., 2015; Tunon et al., 2014). Galectin‐3 inhibition was found to be associated with a reduction in atherosclerotic plaque (Papapanagiotou, Siasos, Kassi, Gargalionis, & Papavassiliou, 2015). Furthermore, modified citrus pectin, a competitive inhibitor for natural ligands of galectin‐3, inhibited cancer progression (Wang & Guo, 2016), inhibited adipose tissue inflammation in obese mice (Martinez‐Martinez et al., 2016), and decreased atherosclerosis in a mouse model of atherosclerosis (MacKinnon et al., 2013). In addition, GR‐MD‐02, a complex carbohydrate‐based drug that binds to galectin‐3, reduced fibrosis in a murine model of nonalcoholic steatohepatitis (Traber & Zomer, 2013). These studies suggested that galectin‐3 may pose important roles in several inflammation‐associated diseases.

In a genome‐wide association study (GWAS), the variance of circulating galectin‐3 levels explained by the locus lectin galactose‐binding soluble 3 (*LGALS3*) that encodes galectin‐3 was 25.6% (de Boer, Verweij, et al., 2012). *LGALS3* genotypes have been associated with susceptibility to dilated cardiomyopathy (Zhang et al., 2018), frequent respiratory tract infections and vaso‐occlusive crisis in children with sickle cell anemia (Mendonca Belmont et al., 2016), the tumor grade and prognosis of glioma (Chen et al., 2012), and the chemotherapeutic response and prognosis of non‐small cell lung cancer (Wu et al., 2012). In this study, we aimed to investigate the associations between *LGALS3* genotypes, circulating galectin‐3 levels, and inflammatory marker levels in patients with CAD.

## 2 MATERIALS AND METHODS

### 2.1 Subjects

The study population has been previously reported (Hsu et al., 2017). In brief, after the exclusion of patients with either marked hemolysis during blood sampling or the absence of available blood samples for DNA or biomarker analyses, 474 patients with angiographically confirmed CAD were enrolled. All patients provided written informed consent, and the study was approved by the Research Ethics Committee of National Taiwan University Hospital (approval number: 201505038RINA). All clinical data were obtained from the patients’ medical records.

### 2.2 Genomic DNA extraction and genotyping

Genomic DNA was extracted according to a process previously reported (Ko, Hsu, Wu, Teng, & Chou, 2016). Three *LGALS3* single‐nucleotide polymorphism (SNP) rs2274273, rs4644, rs4652, which were significantly associated with galectin‐3 levels (*p* < 5 × 10^−8^) in a genome‐wide association study (de Boer, Verweij, et al., 2012) were recruited for the analysis (Table S1). Genotyping of *LGALS3* SNPs was conducted using polymerase chain reaction, followed by TaqMan SNP genotyping assays, which were obtained from Applied Biosystems.

### 2.3 Laboratory examinations and assays

The laboratory examinations and assays were performed as described previously (Hsu et al., 2017). Biomarkers, namely soluble ICAM1 (sICAM1), soluble E‐selectin (sE‐selectin), and MMP9, were measured using commercially available sandwich enzyme‐linked immunosorbent assay (ELISA) kits (R&D). Circulating plasma levels of C‐reactive protein (CRP) were determined using the particle‐enhanced turbidimetric immunoassay technique (Siemens Healthcare Diagnostics Ltd.). The increase in turbidity that accompanies aggregation is proportional to the CRP concentration.

### 2.4 Statistical analysis

Either the chi‐squared test or chi‐squared test for trend was used to examine differences in categorical data distribution and to compare the allele and genotype frequencies. The clinical characteristics of the continuous variables are expressed as mean ± standard deviation and were tested using the two‐sample *t* test or analysis of variance. When the distribution was strongly skewed, median, and interquartile ranges were given. CRP, sICAM1, sE‐selectin, MMP9, and galectin‐3 levels were logarithmically transformed before analysis to achieve adherence to a normality assumption. A Bonferroni correction for multiple testing was used when laboratory variables subjected to multiple testing were taken into account. A generalized linear model was used to analyze the galectin‐3 levels in relation to predictors of the investigated genotypes and confounders. The multivariable stepwise linear regression analysis was conducted with galectin‐3 levels as dependent variable. In the genetic association study, Hardy–Weinberg equilibrium was assessed using Fisher's exact test. All calculations were performed with standard statistical SPSS (version 18 for Windows, Inc.). Values of *p* < .05 using a two‐sided test were considered statistically significant. Golden Helix SVS Win32 8.6.0 software was used to calculate the linkage disequilibrium (LD) between *LGALS3* SNP genotypes, estimated haplotype frequencies, and association of haplotypes with parameter levels. Missing data were approached with list‐wise deletion.

## 3 RESULTS

### 3.1 Baseline data and genetic association study

The demographic characteristics, clinical profiles, biochemical data, hemogram, and inflammatory marker levels with respect to the *LGALS3* rs2274273, rs4644, rs4652 genotypes of the patients are summarized in Table 1 and Tables S2 and S3. After adjustment for age, sex, body mass index (BMI), and current smoking status and a Bonferroni correction for multiple testing, a significant association of galectin‐3 levels with *LGALS3* rs2274273, rs4644, and rs4652 genotypes was observed in the additive inheritance model (*p* = 2.1 × 10^−24^, 7.0 × 10^−24^ and 5.48 × 10^−6^, respectively), however, there was no significant association between *LGALS3* SNP genotypes and other inflammatory marker (CRP, MMP9, sICAM1, and sE‐selectin) levels. Analysis of the *LGALS3* rs4644, rs4652, and rs2274273 allelic and genotypic frequencies showed that all patients were in Hardy–Weinberg equilibrium. The relationship between the study SNPs was analyzed to determine whether the effects of these genetic variants on parameter concentrations could be explained by LD. A nearly complete LD was observed between *LGALS3* SNPs rs4644 and rs2274273 (*r* ^2^ = .986) while the *r* ^2^ measure of LD between rs4652 and rs2274273, and between rs4652 and rs4644 were .337 and .336, respectively (Figure S1).

**Table 1:** Clinical and biochemical characteristics of patients with CAD according to *LGALS3 rs*2274273 genotypes

Genotype (*N*)	Total (474)	CC (315)	TC (143)	TT (16)	*p*1 value	*p*2 value
Baseline characteristics						
Sex (male/female)	381/93	251/64	116/27	14/2	.44	—
Age (years)	65.7 ± 11.4	65.4 ± 11.5	66.7 ± 10.9	61.3 ± 11.6	.97	—
Body mass index (kg/m^2^)	25.9 ± 4.0	26.0 ± 4.2	25.6 ± 3.6	26.5 ± 3.2	.53	—
Hypertension (%)	78.3	79.4	76.9	68.8	.36	—
Diabetes mellitus (%)	44.1	44.4	42.0	56.3	.71	—
Current smoker (%)	24.1	24.4	23.1	25.0	.76	—
Dyslipidemia (%)	60.1	60.3	58.0	75.0	.66	—
Total cholesterol (mg/dl)	179.4 ± 39.3	180.8 ± 36.7	176.9 ± 44.1	174.3 ± 46.3	.11	—
Triglyceride (mg/dl)	123.0 (89.0–178.0)	127.0 (91.0–183.8)	116.0 (80.5–163.3)	158.0 (117.5–210.8)	.52	—
Fasting plasma glucose (mg/dl)	104.0 (92.0–123.0)	103.0 (93.0–120.0)	104.5 (92.0–123.0)	118.0 (99.0–146.0)	.80	—
Creatinine (mg/dl)	1.4 ± 1.4	1.4 ± 1.5	1.3 ± 1.3	1.1 ± 0.2	.25	—
eGFR	69.6 ± 24.4	69.0 ± 25.7	70.7 ± 22.4	70.1 ± 14.6	.63	—
Hemogram						
Leukocyte count (10^3^/μl)	6.7 ± 2.2	6.6 ± 2.0	6.6 ± 2.5	7.1 ± 2.0	.55	—
Hematocrit (%)	40.8 ± 5.4	40.7 ± 5.7	40.8 ± 4.9	42.2 ± 3.9	.47	—
Platelet count (10^3^/μl)	212.2 ± 61.5	215.5 ± 65.9	205.3 ± 52.5	208.3 ± 35.8	.16	—
Inflammation marker levels						
CRP (mg/L)	2.5 (1.3–4.4)	2.6 (1.3–4.4)	2.3 (1.3–4.1)	2.3 (0.6–5.5)	.82	—
MMP9 (ng/ml)	77.1 (48.3–115.3)	75.5 (49.3–119.1)	76.8 (46.7–111.7)	91.9 (49.3–131.6)	.87	—
sICAM1 (ng/ml)	125.9 (107.5–149.6)	127.4 (106.6–151.4)	123.4 (108.3–147.3)	110.9 (97.1–134.1)	.54	—
sE‐selectin (ng/ml)	10.7 (8.2–14.0)	11.0 (8.1–14.3)	10.5 (8.3–13.3)	10.4 (8.0–12.9)	.33	—
Galectin‐3 (ng/ml)	5.7 (3.1–9.2)	6.8 (4.3–11.0)	3.8 (2.2–6.4)	0.6 (0.4–2.0)	1.05 × 10^−25^	2.1 × 10^−24^

Note

Data are presented as mean ± *SD*, percentage, or median (interquartile range), as appropriate.

*p*1 value: adjusted for age, sex, body mass index, and current smoking status.

*p*2 value: after Bonferroni correction and only significant *p* value presented; Bonferroni correction for multiple testing was used with *α* = 0.002 after the 20 tested laboratory variables were taken into account.

Abbreviations: CAD, coronary artery disease; CRP, C reactive protein; eGFR, estimated glomerular filtration rate; MMP9, matrix metalloproteinase 9; *N*, number of subjects; sE‐selectin, soluble E‐selectin; sICAM1, soluble intercellular adhesion molecule 1.

### 3.2 Association of galectin‐3 levels with clinical parameters and inflammatory marker levels

The associations of galectin‐3 levels with clinical parameters and biochemical factors are presented in Table 2. After adjustment for age and sex and a Bonferroni correction for multiple testing, statistically significant correlations were evident between galectin‐3 levels and leukocyte counts, CRP, sICAM1, and MMP9 levels (*p* = 5.69 × 10^−3^, 1.38 × 10^−3^, .015, and 4.52 × 10^−11^, respectively).

**Table 2:** Association between galectin‐3 and biomarker levels in patients with coronary artery disease

	Unadjusted		Adjusted for age, sex		
	*r*	*p* value	*r*	*p* value	Adjusted *p* value
Anthropology					
Age	.12	.01	.09	.06	—
Body mass index	−.007	.88	.03	.59	—
Blood count					
WBC	.16	.001	.16	3.79 × 10^−4^	5.69 × 10^−3^
Hct (%)	−.14	.002	−.08	.09	—
Platelet counts	.09	.04	.11	.01	—
Glucose metabolism					
Fasting plasma glucose	.09	.07	.08	.11	—
Lipid profiles					
Total cholesterol	.007	.89	−.002	.97	—
Triglyceride	.03	.48	.04	.43	—
Renal function					
Creatinine	.08	.10	.08	.10	—
eGFR	−.09	.04	−.06	.24	—
Inflammation marker					
CRP	.18	6.50 × 10^−5^	.18	9.20 × 10^−5^	1.38 × 10^−3^
sICAM1	.17	2.11 × 10^−4^	.15	.001	.015
sE‐selectin	.12	.007	.13	.006	—
MMP9	.30	5.18 × 10^−11^	.31	3.01 × 10^−12^	4.52 × 10^−11^

Note

Adjusted *p* value: after Bonferroni correction; Bonferroni correction for multiple testing was used with *α* = 0.003 after the 15 tested laboratory, variables were taken into account.

For abbreviations, please refer to Table 1.

Abbreviations: Hct, hematocrit; WBC, white blood cell.

### 3.3 Associations of LGALS3 SNP genotypes/haplotypes with galectin‐3 levels

The associations of *LGALS3* SNP genotypes with galectin‐3 levels are presented in Table 3. The minor allele of *LGALS3* rs4644, rs4652, and rs2274273 was consistently associated with a low level of galectin‐3 in patients with CAD in an additive model (*p* = 3.54 × 10^−25^, 2.74 × 10^−7^, 1.05 × 10^−25^, respectively) and dominant model (*p* = 2.08 × 10^−17^, 6.3 × 10^−5^, 1.38 × 10^−17^, respectively). In the haplotype analysis, haplotypes were inferred to capture possible allelic associations. Three haplotypes (CAC, CCC, and ACT) (≥1% frequency) inferred from three SNPs were found to account for 99.7% of all inferred haplotypes (frequency = 60.0%, 21.3%, 18.4%, respectively). After adjustment for age, sex, BMI, and current smoking status, haplotypes CAC, CCC, and ACT were found to be significantly associated with galectin‐3 levels (*p* = 3.13 × 10^−7^, .001, 2.22 × 10^−25^, respectively) (Table 4).

**Table 3:** Association of *LGALS3* SNP genotypes with Galectin‐3 levels in patients with coronary artery disease

*LGALS3* SNP and Galectin‐3 level	MM	Mm	mm	*β*	*p* value	MM	Mm + mm	*β*	*p* value
rs4644	6.78 (4.27–11.01) (315)	3.78 (2.17–6.46) (141)	0.68 (0.43–2.35) (17)	−0.353	3.54 × 10^−25^	6.78 (4.27–11.01) (315)	3.46 (1.87–6.1) (158)	−0.345	2.08 × 10^−17^
rs4652	6.64 (4.30–10.9) (169)	5.30 (3.02–8.52) (220)	3.71 (1.48–6.66) (75)	−0.148	2.74 × 10^−7^	6.64 (4.30–10.9) (169)	5.04 (2.66–8.39) (295)	−0.167	6.3 × 10^−5^
rs2274273	6.8 (4.3–11.0) (315)	3.8 (2.2–6.4) (143)	0.6 (0.4–2.0) (16)	−0.359	1.05 × 10^−25^	6.8 (4.3–11.0) (315)	3.4 (1.9–6.1) (159)	−0.346	1.38 × 10^−17^

Note

*N*, number of subjects.

*p* value: adjusted for age, sex, body mass index, and current smoking status.

Abbreviation: SNP, single‐nucleotide polymorphism.

**Table 4:** Associations of the *LGALS3* haplotypes with circulation levels of Galectin‐3

*LGALS3* haplotypes	Frequency	Coefficient	*p* value
CAC	0.600	0.294	3.13 × 10^−7^
CCC	0.213	0.224	1.09 × 10^−3^
ACT	0.184	−0.724	2.22 × 10^−25^

Note

*p* value: adjusted for age, sex, body mass index, and current smoking status.

Haplotype: rs4644, rs4652, and rs2274273.

### 3.4 Multivariate analysis of parameters influencing galectin‐3 levels

A multivariable stepwise linear regression analysis was used to test the association between galectin‐3 levels and *LGALS3* SNP genotypes while controlling variables of age, sex, BMI, and smoking. The associations between galectin‐3 levels and *LGALS3* rs2274273, rs4644 genotypes remained statistically significant after adjusting these variables (*p* = 1.32 × 10^−19^ and 2.12 × 10^−19^, respectively), and both accounted for 20.8% of the total variance in galectin‐3 levels in this population. However, there is no significant association between galectin‐3 levels and *LGALS3* rs4652 genotypes in the multivariable analysis, which indicates that *LGALS3* rs4652 genotypes is not an independent variable in the association with galectin‐3 levels (Table 5).

**Table 5:** Galectin‐3 levels: stepwise linear regression analysis

	rs4644, rs4652			rs4652, rs2274273		
	*β*	*R* ^2^	*p* value	*β*	*R* ^2^	*p* value
Age	0.004	.008	0.022	0.004	.014	0.016
SEX	0.111	.015	0.02	0.109	.008	0.022
rs2274273	—	—	—	−0.377	.208	1.32 × 10^−19^
rs4644	−0.376	.208	2.12 × 10^−19^	—	—	—
rs4652	0.022	—	0.477	0.023	—	0.46
BMI	0.002	—	0.672	0.002	—	0.648
Smoking	0.005	—	0.9.18	0.005	—	0.914

Note

Multiple linear regression, adjusted for age, sex, smoking status, BMI, and *LGALS3* SNP genotypes.

Abbreviations: BMI, body mass index; SNP, single‐nucleotide polymorphism.

## 4 DISCUSSION

This study analyzed the crucial roles of *LGALS3* variants, galectin‐3 levels, and inflammatory marker levels in a Taiwanese population with CAD. Analysis of the data revealed a significant association between *LGALS3* SNP genotypes and galectin‐3 levels in patients with CAD. We also identified the first evidence that galectin‐3 levels, but not *LGALS3* genotypes, are significantly associated with multiple inflammatory marker levels. To the best of our knowledge, our work is the first study to evaluate the genetic determinants of circulating galectin‐3 levels in patients with CAD. The absence of association between *LGALS3* genotypes and multiple inflammatory marker levels may also provide an important clue that further study is needed to understand the potential role of galectin‐3 for chronic low‐grade inflammation in CAD.

### 4.1 Association between circulating galectin‐3 levels and inflammatory marker levels

In this study, we found statistically significant associations between galectin‐3 levels and multiple inflammatory marker levels. Statistically significant associations between galectin‐3 levels and CRP levels have been reported in patients with diabetes mellitus, cardiac syndrome X, interstitial lung disease, and heart failure (Bozcali et al., 2014; Jin et al., 2013; Srivatsan, George, & Shanmugam, 2015; Zhang, Sun, Song, Zuo, & Xiao, 2014). Our results further demonstrate a similar trend in patients with angiographically confirmed CAD. Colnot et al. (1998) reported that maintenance of granulocyte numbers during acute peritonitis was defective in galectin‐3‐null mice, suggesting galectin‐3 participated in controlling the resolution of acute inflammation. Similar to our results for patients with CAD, Winter et al. (2016) demonstrated a statistically significant correlation between the circulating galectin‐3 level and leukocyte count in patients with premature myocardial infarction. Eosinophil derived from galectin‐3‐deficient mice exhibited considerably reduced rolling on vascular cell adhesion molecule 1 and decreased stable adhesion on ICAM1 under flow conditions in vitro (Ge, Ha, Liu, Rao, & Sriramarao, 2013). Chen et al. (2013) determined that pathological concentrations of galectin‐3 in patients with cancer increased the expression of endothelial cell surface adhesion molecules such as E‐selectin and ICAM‐1, which resulted in enhanced endothelial cell activities in metastasis. In patients with colorectal cancer, Chen et al. (2013) also found that high serum galectin‐3 levels were statistically significantly correlated with high serum sICAM1 concentrations. In this investigation, our data revealed a statistically significant positive association between circulating galectin‐3 levels and sICAM‐1 and a trend of association between galectin‐3 levels and sE‐selectin in patients with CAD. The interaction between galectin‐3 and MMP9 was more complex. MMPs, including MMP2 and MMP9, have been determined to be capable of effectively cleaving galectin‐3, thereby modulating the biological function of galectin‐3; consequently, galectin‐3 acts as a downstream regulator of MMP9 function (Ochieng et al., 1994; Ochieng, Green, Evans, James, & Warfield, 1998; Ortega, Behonick, Colnot, Cooper, & Werb, 2005). Galectin cleavage was demonstrated to be an active process during tumor progression and can be used as a novel surrogate marker for MMP activity in growing breast cancers (Nangia‐Makker et al., 2007). By contrast, galectin‐3 facilitated cell motility in cancer cells by inducing MMPs, including MMP9 (Kim et al., 2011; Mauris, Woodward, Cao, Panjwani, & Argueso, 2014; Wang et al., 2012), and galectin‐3 gene silencing considerably suppressed the mRNA and protein levels of MMP9 (Zhang et al., 2012). One study suggested that MMP9 expression may be induced by galetin‐3 activating the p38 MAPK pathway (Dange et al., 2015). Thus, bidirectional interactions exist between galectin‐3 and MMP9, highlighting the complexity of the associations between galectin‐3 and MMP9. All the aforementioned results suggested that galectin‐3 may participate in multiple inflammation processes through a molecular mechanism relating to its interaction with these inflammatory biomarkers.

### 4.2 Association between LGALS3 genotypes and circulating galectin‐3 levels

In a GWAS of Caucasian individuals, de Boer, Verweij, et al. (2012)) discovered that rs2274273 was the lead SNP in the *LGALS3* locus for circulating galectin‐3 levels in a relatively healthy population. Considerably lower galectin‐3 levels have also been associated with the rare allele of the *LGALS3* genotypes, including rs4644, rs4652, and rs2274273, in populations with diseases such as rheumatoid arthritis, sickle cell anemia, and dilated cardiomyopathy (Hu, Chang, Wu, Tsai, & Hsu, 2011; Mendonca Belmont et al., 2016; Zhang et al., 2018). Consistent with these results, we further demonstrated that the T variant of *LGALS3* rs2274273 genotypes, the A variant of *LGALS3* rs4644 genotypes and the C variant of *LGALS3* 4,652 genotypes were associated with relatively low galectin‐3 levels in patients with CAD, and the effect size was similar to that evident in the general population, as reported by Boer et al. (de Boer, Verweij, et al., 2012). However, Djordjevic et al. found considerably high expression of *LGALS3* in the carotid plaque tissues of rs2274273 rare allele carriers with advanced atherosclerosis (Djordjevic et al., 2016). A possible explanation for this discrepancy is that mRNA and protein levels may not correlate well (Maier, Guell, & Serrano, 2009). Another possibility is that the sample size was relatively small in Djordjevic's study, yielding only borderline significance for the association. However, the genetic association study mentioned earlier should also be interpreted with caution. Research suggested that the two antibodies in the ELISA assay of galectin‐3 recognize epitopes near the region of the two nonsynonymous variants *LGALS3* rs4644 and rs4652, which are in strong LD with rs2274273 and may cause artifactual results of the association between these *LGALS3* SNP genotypes and galectin‐3 levels (de Boer, Verweij, et al., 2012). In our study, significant associations between these *LGALS3* SNP genotypes and galectin‐3 levels were evident. The immunogen for the human galectin‐3 antibody used in our ELISA assay was recombinant human galectin‐3 Ala2‐Ile250, which represented near whole length of human galectin‐3. The minor allele of both rs4644 and rs4652 were significantly associated with lower galectin‐3 level (0.68 ng/ml and 3.71 ng/ml, respectively). However, the LD between rs4644 and rs4652 was not strong (*r* ^2^ = .336). These findings indicated that both missense variant rs4644 and rs4652 has significant but different potency on galectin‐3 level. In a Brazil study with mixed‐race ancestry (European, African and Amerindian Contribution), the serum galectin‐3 levels were associated with rs4644 and rs4652 genotypes and the distribution of serum galectin‐3 levels according to additive model were similar to our results. The rs4644 CC was associated with higher galectin‐3 levels, followed by CA and finally the AA genotype and rs4652 AA genotype was associated with higher levels, followed by AC and CC genotype (Mendonca Belmont et al., 2016). These similar distribution of serum galectin‐3 levels according to additive model derived from different ELISA kit in different racial population and the significant but different potency of rs4644 and rs4652 variants on galectin‐3 level demonstrated in our data determined by the ELISA antibody binding the immunogen which represented near whole length of human galectin‐3 may lower the possibility of artifactual results of the association secondary to the effect mentioned by de Boer, Verweij, et al. (2012)). These evidences and our results suggest that *LGALS3* SNP genotypes are significantly associated with circulating galectin‐3 levels and may further affect the inflammatory process of various inflammation‐related diseases.

### 4.3 The role of galectin‐3 in the association between LGALS3 genotypes and inflammation marker levels

Our results demonstrated that not only inflammatory marker levels, but also genetic variants of *LGALS3* were independently associated with galectin‐3 levels in patients with CAD. The strong association between *LGALS3* *g*enotypes and galectin‐3 levels suggested that *LGALS3* SNPs could act as an instrumental variable for causal relationship analysis. However, there was no significant association between *LGALS3* SNP genotypes and other inflammatory marker (CRP, MMP9, sICAM1, and sE‐selectin) levels. Thus, the finding that galectin‐3 levels are associated with inflammatory marker levels could have been the result of confounding or reversed causality in this group of CAD patients. As mentioned above, there were complex interactions between galectin‐3 and inflammation markers, such as the bidirectional effect between galectin‐3 and MMP9. Our results suggested that galectin‐3 may not play a causative role, but may act as a surrogate marker, in many processes of low‐grade inflammation in CAD.

### 4.4 Association between LGALS3 genotypes rs4644, rs4652 and rs2274273

In this study, our results showed a strong LD between rs4644 and rs2274273 (*r* ^2^ = .986) but not between rs4652 and rs2274273/rs4644 (*r* ^2^ = .337 and 0.336, respectively). In a China study, rs4644 has high LD with rs2274273 (*r* ^2^ = .974) (Zhang et al., 2018). In another report evaluating the *LGALS3* genotypes rs4644 and rs4652 in Chinese patients with NSCLC, LD between these two polymorphisms was not observed (Wu et al., 2012). However, in studies focus on European population, high LD between rs4644, rs4652, and rs2274273 (de Boer, Verweij, et al., 2012) and high LD between rs4644 and rs4652 (Trompet et al., 2012) had been reported. The minor allele frequency of *LGALS3* genotypes rs4644, rs4652, and rs2274273 for this sample population were 19%, 39.9%, and 19%, respectively which were comparable to that reported for other Han Chinese groups (National Center for Biotechnology Information. dbSNP. HapMap‐HCB; Zhang et al., 2018). However, the minor allele frequency for rs4644, rs4652, and rs2274273 in European population were 40%–49%, 42%–49%, and 41%–49.1%, respectively (de Boer, Verweij, et al., 2012; National Center for Biotechnology Information. dbSNP. HapMap‐CEU; Trompet et al., 2012). These findings indicated a possibility of racial difference between European and Han Chinese population.

### 4.5 Limitations

This study has several limitations. The first limitation is its modest sample size. Replication of this investigation using a larger sample cohort would improve the strength of the analysis. In addition, the cross‐sectional design of this study limits our ability to infer causal relationships between *LGALS3* genotypes, galectin‐3 levels, and inflammatory marker levels. Finally, all the patients in the study were ethnically Chinese; therefore, caution should be exercised when extrapolating our results to other ethnic groups.

## 5 CONCLUSION

The results in this investigation revealed that both *LGALS3* genotypes and inflammatory marker levels play a crucial role in determining the circulating galectin‐3 levels in patients with CAD. The absence of a significant association between *LGALS3* gene variants and the inflammation markers levels suggested that it should be cautious when galectin‐3 was used as a therapeutic target for chronic inflammatory disorders, such as CAD. Complex correlations between *LGALS3* gene variants, galectin‐3 levels, and inflammation marker levels also imply that further studies involving populations from different ethnic groups and disease status may be necessary to elucidate the definite role of galectin‐3 in various inflammatory‐related disorders.

## CONFLICT OF INTEREST

The authors declare that they have no conflicts of interest.

## AUTHORS' CONTRIBUTIONS

YHL, JMJJ, and YLK designed the study. YHL and YLK analyzed the data and wrote the manuscript. MST and SW performed several experiments and helped in analysis of the data and writing the manuscript. JMJJ and FTC participated in preparation of the blood samples for genotyping and collection of the clinical data. LKE helped in discussing and writing the manuscript. All authors read and approved the final manuscript.

## ETHICS APPROVAL AND CONSENT TO PARTICIPATE

The study was approved by the Research Ethics Committee of National Taiwan University Hospital (approval number: 201505038RINA). All patients provided written informed consent.

## Supporting information

Fig S1

Table S1‐S3

## Data Availability

The data used to support the findings of this study are included in the article and the supplementary material of this article.
