# Supplementary figures and images for: Genetic determinants of circulating galectin‐3 levels in patients with coronary artery disease
Source: Mol Genet Genomic Med. 2020 Jun 23;8(9):e1370. doi: 10.1002/mgg3.1370 (PMC7507567; doi:10.1002/mgg3.1370)

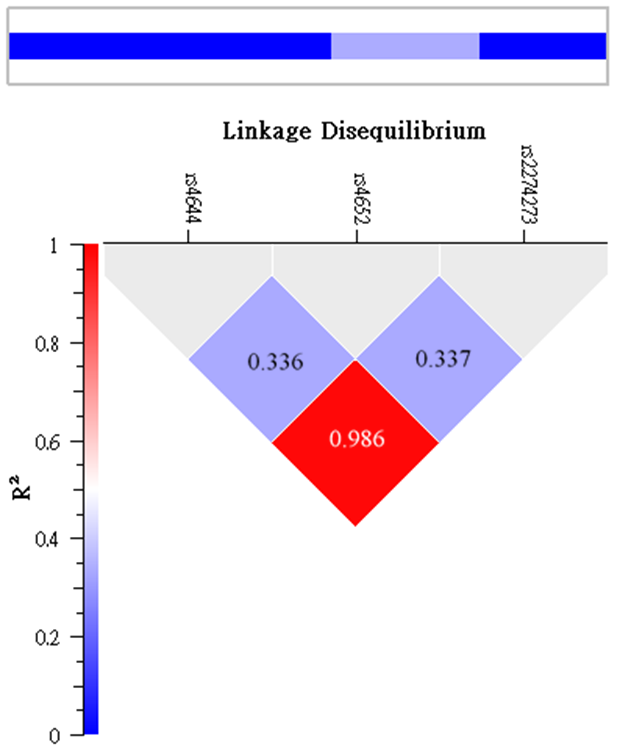

Supplement: Supplementary file 1 — Fig S1 [file MGG3-8-e1370-s001.tif]
