# Supplementary material for: Genetic determinants of circulating galectin‐3 levels in patients with coronary artery disease
Source: Mol Genet Genomic Med. 2020 Jun 23;8(9):e1370. doi: 10.1002/mgg3.1370 (PMC7507567; doi:10.1002/mgg3.1370)
Supplement: Supplementary file 2 — Table S1‐S3 [file MGG3-8-e1370-s002.docx]

Supplementary Table 1. *LGALS3* SNPs

| SNP number | Chromosome | Position | Gene | Locaction | Genotype | MAF | SNP Function |
| --- | --- | --- | --- | --- | --- | --- | --- |
| rs4644 | 14 | 55138217 | LGALS3 | 9000 | A/C | 0.185 | Missense |
| rs4652 | 14 | 55138318 | LGALS3 | 9101 | A/C | 0.399 | Missense |
| rs2274273 | 14 | 55147918 | LGALS3 | 18701 | C/T | 0.185 | 3' |
|  |  |  | DLGAP5 | (3')198 |  |  | 3’UTR 500B |

SNP: single nucleotide polymorphism; MAF: minor allele frequency

Supplementary table 2. Clinical and biochemical characteristics of patients with CAD according to LGALS3 rs4644 genotypes

| Genotype (N) | Total (473) | CC (315) | CA (141) | AA(17) | *P1 value* | *P2 value* |
| --- | --- | --- | --- | --- | --- | --- |
| Baseline characteristics |  |  |  |  |  |  |
| Sex (male/female) | 380/93 | 251/64 | 114/27 | 15/2 | 0.488 | - |
| Age (years) | 65.7 ± 11.4 | 65.5 ± 11.6 | 66.6 ± 10.9 | 61.8 ± 11.5 | 0.930 | - |
| Body mass index (kg/m^2^) | 25.9 ± 4.0 | 26.0 ± 4.1 | 25.6 ± 3.6 | 26.6 ± 3.2 | 0.546 | - |
| Hypertension (%) | 78.4 | 79.7 | 76.6 | 70.6 | 0.309 | - |
| Diabetes mellitus (%) | 44.2 | 44.4 | 42.6 | 52.9 | 0.729 | - |
| Current smoker (%) | 24.1 | 24.4 | 23.4 | 23.5 | 0.778 | - |
| Dyslipidemia (%) | 60.2 | 60.6 | 57.4 | 76.5 | 0.712 | - |
| Total cholesterol (mg/dl) | 179.4 ± 39.4 | 180.8 ± 36.7 | 176.6 ± 44.3 | 175.0 ± 44.9 | 0.109 | - |
| Triglyceride (mg/dl) | 123.0 (89.0 – 177.5) | 127.0 (91.0 - 182.8) | 116.0 (79.5 - 162.8) | 165.0 (118.0 - 252.5) | 0.685 | - |
| Fasting plasma glucose (mg/dl) | 104.0 (92.5 - 123.0) | 103.0 (93.0 - 120.0) | 105.0 (92.0 - 123.0) | 111.5 (98.3 - 144.5) | 0.442 | - |
| Creatinine (mg/dl) | 1.4 ± 1.4 | 1.4 ± 1.5 | 1.3 ± 1.3 | 1.1 ± 0.2 | 0.252 | - |
| eGFR | 69.5 ± 24.4 | 69.0 ± 25.7 | 70.7 ± 22.5 | 70.1 ± 14.2 | 0.653 | - |
| Hemogram |  |  |  |  |  |  |
| Leukocyte count (10^3^/μL) | 6.7 ± 2.2 | 6.6 ± 2.0 | 6.6 ± 2.6 | 7.0 ± 2.0 | 0.556 | - |
| Haematocrit (%) | 40.7 ± 5.4 | 40.7 ± 5.7 | 40.7 ± 4.9 | 42.3 ± 3.8 | 0.475 | - |
| Platelet count (10^3^/μL) | 212.3 ± 61.5 | 215.5 ± 65.9 | 206.2 ± 52.3 | 203.6 ± 39.5 | 0.130 | - |
| Inflammation marker levels |  |  |  |  |  |  |
| CRP (mg/L) | 2.5 (1.3 - 4.4) | 2.6 (1.3 - 4.5) | 2.3 (1.3 - 4.0) | 2.2 (0.6 - 5.3) | 0.672 | - |
| MMP9 (ng/mL) | 76.8 (48.3 - 115.3) | 74.9 (49.0 - 119.1) | 78.1 (46.9 - 111.9) | 91.4 (52.7 - 122.8) | 0.961 | - |
| sICAM1 (ng/mL) | 126.2 (107.6 - 149.9) | 127.4 (106.8 - 151.4) | 123.4 (108.3 - 147.6) | 111.6 (98.5 - 133.8) | 0.521 | - |
| sE-selectin (ng/mL) | 10.7 (8.2 - 14.0) | 11.0 (8.1 - 14.3) | 10.5 (8.4 - 13.2) | 10.8 (8.0 – 13.6) | 0.382 | - |
| Galectin-3 (ng/mL) | 5.7 (3.1 - 9.2) | 6.8 (4.3 - 11.0) | 3.8 (2.2 - 6.5) | 0.7 (0.4 - 2.4) | 3.5 × 10^-25^ | 7.0 × 10^-24^ |

For abbreviations, please refer to Table1

*P1* value: adjusted for age, sex, body mass index, and current smoking status.

*P*2 value: after Bonferroni correction and only significant *P* value presented; A Bonferroni correction for multiple testing was used with α = 0.002 after the 20 different tested laboratory variables were taken into account.

Supplementary table 3. Clinical and biochemical characteristics of patients with CAD according to LGALS3 rs4652 genotypes

| Genotype (N) | Total (474) | AA (169) | AC (220) | CC (75) | *P1 value* | *P2 value* |
| --- | --- | --- | --- | --- | --- | --- |
| Baseline characteristics |  |  |  |  |  |  |
| Sex (male/female) | 381/93 | 134/35 | 175/45 | 65/10 | 0.367 | - |
| Age (years) | 65.7 ± 11.4 | 66.1 ± 11.7 | 65.7 ± 11.2 | 63.4 ± 10.8 | 0.172 | - |
| Body mass index (kg/m^2^) | 25.9 ± 4.0 | 25.8 ± 4.5 | 25.9 ± 3.8 | 26.3 ± 3.1 | 0.587 | - |
| Hypertension (%) | 78.3 | 79.9 | 77.7 | 74.7 | 0.309 | - |
| Diabetes mellitus (%) | 44.1 | 50.3 | 41.4 | 42.7 | 0.193 | - |
| Current smoker (%) | 24.1 | 26.0 | 23.2 | 22.7 | 0.233 | - |
| Dyslipidemia (%) | 60.1 | 59.2 | 56.4 | 73.3 | 0.126 | - |
| Total cholesterol (mg/dl) | 179.4 ± 39.3 | 180.8 ± 36.2 | 178.6 ± 41.19 | 177.3 ± 39.9 | 0.415 | - |
| Triglyceride (mg/dl) | 123.0 (89.0 - 178.0) | 127.0 (87.3 - 191.8) | 121.0 (89.0 - 166.0) | 131.0 (96.0 - 189.0) | 0.714 | - |
| Fasting plasma glucose (mg/dl) | 104.0 (92.0 - 123.0) | 105.5 (92.8 - 125.3) | 102.0 (92.0 - 118.5) | 106.0 (93.0 - 131.0) | 0.734 | - |
| Creatinine (mg/dl) | 1.4 ± 1.4 | 1.5 ± 1.8 | 1.4 ± 1.4 | 1.1 ± 0.3 | 0.065 | - |
| eGFR | 69.6 ± 24.4 | 68.7 ± 26.5 | 68.9 ± 24.5 | 73.3 ± 19.0 | 0.484 | - |
| Hemogram |  |  |  |  |  |  |
| Leukocyte count (10^3^/μL) | 6.7 ± 2.2 | 6.6 ± 2.0 | 6.5 ± 1.8 | 7.3 ± 3.2 | 0.036 | - |
| Haematocrit (%) | 40.8 ± 5.4 | 40.2 ± 5.9 | 40.7 ± 5.2 | 42.0 ± 4.5 | 0.114 | - |
| Platelet count (10^3^/μL) | 212.2 ± 61.5 | 215.7 ± 73.1 | 206.2 ± 54.1 | 226.2 ± 52.5 | 0.668 | - |
| Inflammation marker levels |  |  |  |  |  |  |
| CRP (mg/L) | 2.5 (1.3 - 4.4) | 2.8 (1.2 - 4.9) | 2.3 (1.3 - 4.2) | 2.3 (1.1 - 4.3) | 0.694 | - |
| MMP9 (ng/mL) | 77.1 (48.3 - 115.3) | 77.5 (50.4 - 125.5) | 73.9 (46.6 - 112.4) | 82.7 (48.3 - 122.1) | 0.764 | - |
| sICAM1 (ng/mL) | 125.9 (107.5 - 149.6) | 128.5 (107.1 - 154.0) | 123.4 (107.6 - 149.1) | 125.4 (107.5 - 142.9) | 0.293 | - |
| sE-selectin (ng/mL) | 10.7 (8.2 - 14.0) | 11.0 (8.2 - 14.4) | 10.8 (8.3 - 13.9) | 10.7 (7.7 - 13.3) | 0.370 | - |
| Galectin-3 (ng/mL) | 5.7 (3.1 - 9.2) | 6.6 (4.3 - 10.9) | 5.3 (3.0 - 8.5) | 3.7 (1.5 – 6.7) | 2.74 × 10^-7^ | 5.48 × 10^-6^ |

For abbreviations, please refer to Table1

*P1* value: adjusted for age, sex, body mass index, and current smoking status.

*P*2 value: after Bonferroni correction and only significant *P* value presented; A Bonferroni correction for multiple testing was used with α = 0.002 after the 20 different tested laboratory variables were taken into account.
